# Moose movement rates are altered by wolf presence in two ecosystems

**DOI:** 10.1002/ece3.4402

**Published:** 2018-08-19

**Authors:** Mark A. Ditmer, John R. Fieberg, Ron A. Moen, Steve K. Windels, Seth P. Stapleton, Tara R. Harris

**Affiliations:** ^1^ Conservation Department Minnesota Zoo Apple Valley Minnesota; ^2^ Department of Fisheries, Wildlife, and Conservation Biology University of Minnesota St. Paul Minnesota; ^3^ Department of Biology Natural Resources Research Institute University of Minnesota Duluth Duluth Minnesota; ^4^ Voyageurs National Park International Falls Minnesota

**Keywords:** behavioral modifications, first‐passage time, habitat selection, movement ecology, predation risk, predator–prey interactions

## Abstract

Predators directly impact prey populations through lethal encounters, but understanding nonlethal, indirect effects is also critical because foraging animals often face trade‐offs between predator avoidance and energy intake. Quantifying these indirect effects can be difficult even when it is possible to monitor individuals that regularly interact. Our goal was to understand how movement and resource selection of a predator (wolves; *Canis lupus*) influence the movement behavior of a prey species (moose; *Alces alces*). We tested whether moose avoided areas with high predicted wolf resource use in two study areas with differing prey compositions, whether avoidance patterns varied seasonally, and whether daily activity budgets of moose and wolves aligned temporally. We deployed GPS collars on both species at two sites in northern Minnesota. We created seasonal resource selection functions (RSF) for wolves and modeled the relationship between moose first‐passage time (FPT), a method that discerns alterations in movement rates, and wolf RSF values. Larger FPT values suggest rest/foraging, whereas shorter FPT values indicate travel/fleeing. We found that the movements of moose and wolves peaked at similar times of day in both study areas. Moose FPTs were 45% lower in areas most selected for by wolves relative to those avoided. The relationship between wolf RSF and moose FPT was nonlinear and varied seasonally. Differences in FPT between low and high RSF values were greatest in winter (−82.1%) and spring (−57.6%) in northeastern Minnesota and similar for all seasons in the Voyageurs National Park ecosystem. In northeastern Minnesota, where moose comprise a larger percentage of wolf diet, the relationship between moose FPT and wolf RSF was more pronounced (ave. across seasons: −60.1%) than the Voyageurs National Park ecosystem (−30.4%). These findings highlight the role wolves can play in determining moose behavior, whereby moose spend less time in areas with higher predicted likelihood of wolf resource selection.

## 1 INTRODUCTION

Predators affect prey populations directly through lethal encounters and indirectly through behavioral modifications that reduce encounter rates (Peckarsky et al., 2008). Antipredator decisions involve an intrinsic trade‐off between safety and foraging efficiency (Brown & Kotler, 2004; Lima & Dill, 1990). Although some species and demographic groups can avoid or mitigate some of the impacts of predation risk (Fortin, Boyce, Merrill, & Fryxell, 2004; Laundré, Hernández, & Altendorf, 2001; Wolff & Horn, 2003), both theoretical and field studies have linked antipredator behavior to changes in resource selection (Creel, Winnie, Maxwell, Hamlin, & Creel, 2005), increased energetic costs resulting from foraging in suboptimal habitat or for less desirable food resources (Creel, Winnie, & Christianson, 2009; Hernández & Laundré, 2005), and increased rates of movement or spatial displacement (Abrahams & Dill, 1989; Fortin et al., 2005). Chronic antipredator behavioral responses have been correlated with higher levels of stress and reduced recruitment (Creel, Christianson, Liley, & Winnie, 2007; Clinchy, Sheriff, & Zanette, 2013; Cherry, Morgan, Rutledge, Conner, & Warren, 2016; although see Boonstra, 2013).

Ignoring the costs of behavioral modifications associated with predation or encounter risk may wrongly attribute prey population changes to alterations in resource quality or quantity, leading to ill‐informed management decisions (Creel et al., 2007). However, quantifying the indirect costs of predation risk can be difficult; prey species not only respond to the current spatial distribution of predators, but they may also avoid areas of the landscape frequented by predators or where attack success is greatest (Hebblewhite, Merrill, McDonald, & Ranta, 2005). Prey commonly become more vigilant in response to an increased likelihood of a predator encounter (Wolff & Horn, 2003), which can increase movement or reduce time spent foraging (Cherry, Conner, & Warren, 2015). Developing better ways to discern behavioral changes of prey species, such as through remote technologies that measure fine‐scale movements, in areas where predators are more likely to be found can help quantify the costs of antipredator behavior.

Wolves can influence the foraging patterns of ungulates (Latombe, Fortin, & Parrott, 2014), including moose (*Alces alces*), a keystone herbivore that affects ecosystem function and structure (Faison, DeStefano, Foster, Motzkin, & Rapp, 2016; Moen, Cohen, & Pastor, 1998; Pastor et al., 1998). Predation by wolves can limit population growth of moose (Bergerud, Wyett, & Snider, 1983), and studies have associated landscape attributes, such as habitat type or distance to shoreline, with higher risk of predation by wolves for moose (Kunkel & Pletscher, 2000; Montgomery, Vucetich, Roloff, Bump, & Peterson, 2014). Discerning the influence of wolves on moose populations living along their southern range is even more critical because moose face an additional trade‐off between habitats with high forage and areas of thermal shelter (van Beest, Van Moorter, & Milner, 2012; Street, Rodgers, & Fryxell, 2015; Street et al., 2016). Altering behavior to avoid or pass quickly through areas of the landscape where wolves are more likely to be present may reduce foraging or cooling opportunities.

Several moose populations along the southern geographic extent of their range have exhibited declines in abundance and/or compromised health (Dou, Jiang, Stott, & Piao, 2013; Grøtan, Sæther, Lillegård, Solberg, & Engen, 2009; Monteith et al., 2015; Murray et al., 2006; Ruprecht et al., 2016), while others appear to be healthy (Brimeyer & Thomas, 2004; Murray et al., 2012; Wattles, Zeller, & DeStefano, 2018). In northeastern Minnesota (NEMN), which is high‐quality moose habitat, moose declined 50% in abundance over the last decade (2005 to 2016; DelGiudice, 2016; ArchMiller, Dorazio, St. Clair, & Fieberg, 2018). A growing wolf population that has benefited from legal protection and the seasonal availability of deer is a primary driver in the decline of moose in NEMN (Barber‐Meyer & Mech, 2016; Mech & Fieberg, 2014; Mech, Fieberg, & Barber‐Meyer, 2018). The deer population in this region provides an additional prey item for wolves during part of the year and may help sustain a larger wolf population, yet dietary estimates and calf predation studies suggest wolves here still regularly prey on moose in NEMN (Chenaux‐Ibrahim, 2015; Severud et al., 2015). In contrast, wolves in Minnesota's Voyageurs National Park (VNP) ecosystem consume far less moose, instead preying on deer and American beavers (*Castor canadensis*; Gogan et al., 2004; Chenaux‐Ibrahim, 2015; Gable, Windels, Bruggink, & Homkes, 2016; Gable, Windels, & Bruggink, 2017; Gable, Windels, & Olson, 2017). In VNP, both moose and wolf population numbers have been stable for decades (Windels & Olson, 2016).

Here, we quantify the nonlethal, indirect effects of wolves on moose movement behavior using GPS locations from both collared wolves and moose inhabiting NEMN and VNP ecosystems. We aim to measure the changes in moose behavior as a function of the predicted likelihood of wolf resource selection. In particular, we test (a) if moose movements were altered in areas highly selected by wolves, (b) if moose in NEMN, where moose comprise a larger percentage of wolf diet, show increased movement rates in high‐predation‐risk areas compared to moose in VNP, (c) if the relationship between moose behavior and predicted wolf resource selection varies across seasons when moose may be more or less susceptible to attack, and (d) if moose are most active during the same times of day as wolves. Our analysis connects the behavioral changes in the movements of a prey species across gradients in predator resource selection probability. In doing so, we provide new insights into the impacts of wolf presence on the movements of a vulnerable moose population.

## 2 MATERIALS AND METHODS

### 2.1 Study areas

**Figure 1:**
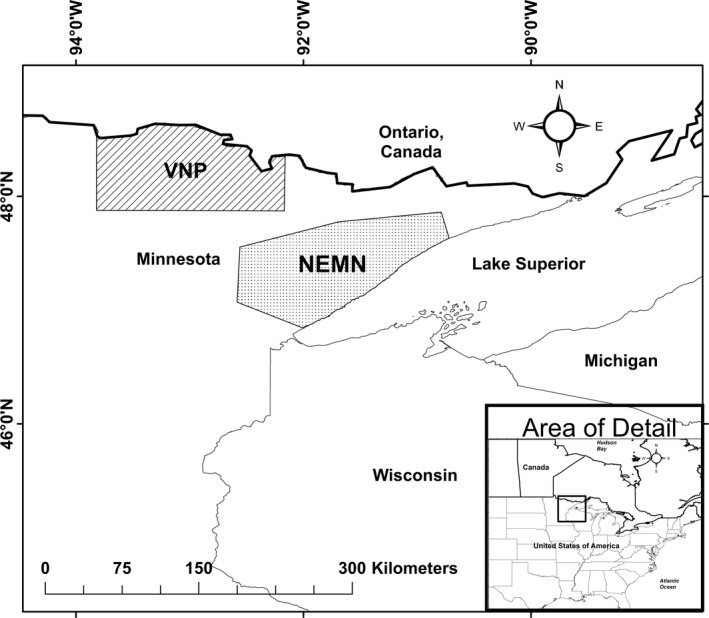
Study areas in northeastern Minnesota (NEMN) and the Voyageurs National Park (VNP) ecosystem where we studied the influence of wolf presence on moose behavior using GPS‐collared individuals of both species from 2011 to 2015

Our study areas in NEMN and VNP are both located in northern Minnesota (Figure 1), which has a mid‐continental climate with moderate precipitation. Summers are short and warm (average daily July [warmest month] temperatures; NEMN = 18.8°C; VNP = 18.9°C), while snow cover typically occurs from November through March with cold temperatures (average daily January [coldest month] temperatures; NEMN = −10.7°C; VNP = −13.1°C). The average temperatures are based on National Oceanic Atmosphere Administration's National Climatic Data Center ( http://www.ncdc.noaa.gov) from 2006 to 2016 for the Global Climate Station Summary from Duluth (NEMN) and Falls International (VNP) Airports in MN, USA.

NEMN and VNP study areas contain numerous lakes and ponds, but VNP has more topographic relief with rocky outcrops and shoreline bluffs. Aside from a few rocky outcrops around lakes, the NEMN study area gently slopes toward the northern shore of Lake Superior which marks the eastern edge of the study area. Northern Minnesota includes the transition zone between the Canadian boreal forests and northern hardwood forests (Pastor & Mladenoff, 1992). Overall, NEMN and VNP are primarily a mix of forest (% areal coverage of deciduous, conifer, and mixed forest types; NEMN = 46%; VNP = 39%) and woody wetlands (NEMN = 33%; VNP = 37%). Forest canopy species in VNP consist of aspen (*Populus* spp.), paper birch (*Betula papyrifera*), balsam fir (*Abies balsamea*), spruce (*Picea* spp.), pine (*Pinus* spp.) and red maple (*Acer rubrum*; Faber‐Langendoen, Aaseng, Hop, Lew‐Smith, & Drake, 2007). NEMN overstory is dominated by aspen, white spruce (*Picea glauca*), and paper birch. Within the National Park boundaries of the VNP study area, which encompasses the roadless Kabetogama Peninsula (305 km^2^), there is only natural forest disturbance and development is limited to maintained hiking trails in the summer and snowmobile trails during the winter months. However, throughout the larger VNP study area and NEMN, forest management for timber has resulted in regenerating aspen and jack (*Pinus banksiana*) and red (*P. resinosa*) pine plantations. Woody wetland species are primarily alder (*Alnus* spp.), black spruce (*Picea mariana*), and cedar (*Thuja occidentalis*) in NEMN and tamarack (*Larix laricina*) and black ash (*Fraxinus nigra*) in the VNP ecosystem. The majority of the remaining land cover types consist of open water (NEMN = 6%; VNP = 9%), herbaceous wetlands (NEMN = 4%; VNP = 7%), and shrub/scrub (NEMN = 8%; VNP = 6%).

The relative density of moose in NEMN (2016 estimate: $\overset{¯}{X}$ = ~0.26 [90% CI = ~0.21–0.34] moose/km^2^; DelGiudice, 2016) exceeds that of VNP (~0.13 [90% CI = ~0.13–0.15] moose/km^2^ in the Kabetogama Peninsula, <0.05 elsewhere in study area; Windels & Olson, 2016). There is no harvest of moose in the VNP ecosystem (inside or outside of the park). The state of Minnesota's moose hunt in NEMN was suspended indefinitely in 2012 due to concern for the declining population. Wolf densities in VNP were estimated at 4–6/100 km^2^ (Gable et al., 2016). There is no estimate of wolf density specific to the NEMN area, but the average for the greater MN wolf range was 3.2/100 km^2^ (includes NEMN in average) with a total population estimate of ~2,500 individuals (Erb, Humpal, & Sampson, 2015). Wolves in NEMN have been afforded legal protection since 1975 aside from a harvest during 2012–2014 (413 and 238 individuals harvested in 2012 and 2013, respectively; Stark & Erb, 2014). VNP wolves are protected from hunting. Other prey items for wolves, such as white‐tailed deer, are present in both study areas. VNP deer occur at ~2–4 deer/km^2^ (Gable, Windels, & Bruggink, 2017; Gable, Windels, & Olson, 2017) within the National Park and ~2.1 deer/km^2^ in the surrounding area (2015 estimate; D'Angelo & Giudice, 2015). In NEMN, deer density decreases from south (~3.5 deer/km^2^) to north (~1.5 deer/km^2^; 2015 estimates; D'Angelo & Giudice, 2015). Deer density is higher to the east in NEMN along the shores of Lake Superior, but the population is semimigratory; deer move further inland during the winter season (Fieberg, Kuehn, & DelGiudice, 2008; Nelson, 1995). Deer populations in both study areas vary greatly with winter severity and the 2015 estimates provided here mark the lowest density estimates from 2010 to 2015. Another prey item for wolves, beaver, can be found at densities in VNP that may be the highest in the United States (~5.0 beaver/km^2^; Johnston & Windels, 2015), while densities in NEMN are generally much lower (~1.3–2.5 beaver/km^2^; Berg, 2000).

### 2.2 Animal capture and handling

#### 2.2.1 Wolves

We captured adult wolves in VNP (2012 to 2014) and NEMN (2014 to 2015) using padded foothold traps (Livestock Protection Company, Alpine, TX) from June to October. We immobilized wolves with a mixture of ketamine (10 mg/kg) and xylazine (2 mg/kg). We fit individual wolves with either an Argos GPS (Telonics, Inc., Mesa, AZ, USA) or an Iridium GPS collar (Lotek Wireless, Inc., Newmarket, Ontario, Canada; Vectronic Aerospace GmbH, Berlin, Germany). The collars collected GPS locations up to 2 years and attempted a fix once every 20 min−6 hr, but most were every 4−6 hr in VNP and every 2 hr in NE.

#### 2.2.2 Moose

From 2010 to 2012 in VNP and 2011‐2013 in NEMN, we used helicopters to dart and capture moose (Quicksilver Air, Inc., Fairbanks, AK, USA) during February and March. We immobilized individuals with a mixture of carfentanil citrate (1.2 ml; 4.0 mg/ml) and xylazine HCl (1.2 ml; 100 mg/ml). We used naltrexone HCl (7.2 ml; 50 mg/ml) and yohimbine HCl (3 ml; 5 mg/ml) as antagonists. We outfitted moose with global positioning system (GPS) collars in both VNP (Sirtrack Limited, Hawkes Bay, New Zealand) and NEMN (Lotek Wireless, Inc., Newmarket, Ontario, Canada; Vectronic Aerospace GmbH, Berlin, Germany). Fix attempts in VNP were scheduled at 15‐min intervals during 2010 and 20‐min intervals during 2011–2012, while all fix attempts were at 20‐min intervals in NEMN. We estimated the GPS error of locations from stationary Sirtrack collars to be an average of ~7 m for a 50% circular error (McCann, Moen, Windels, & Harris, 2016). All animal capture and handling protocols were approved by the University of Minnesota and National Park Service Animal Care and Use committees.

### 2.3 Analytical methods

#### 2.3.1 Overview

**Figure 2:**
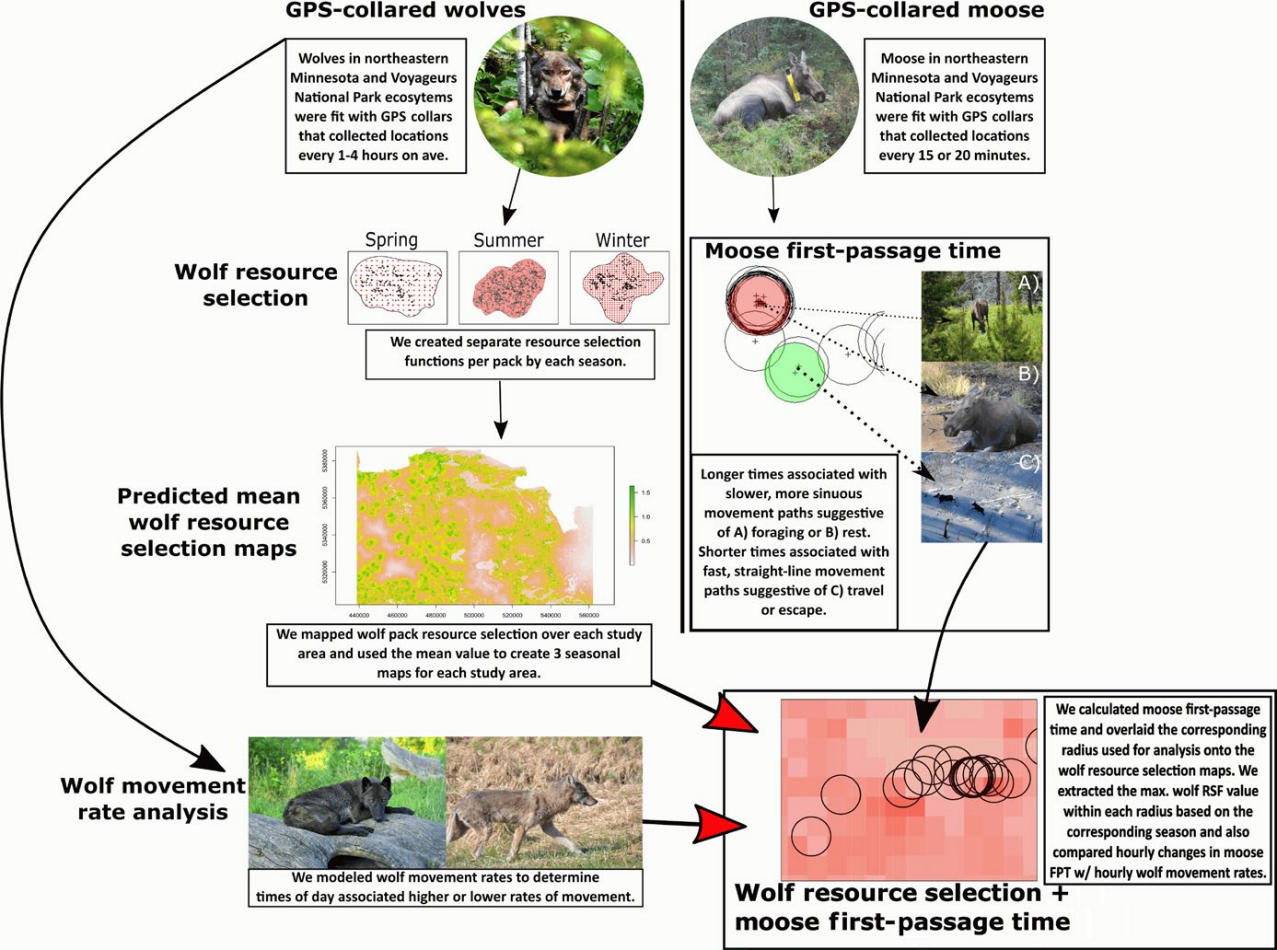
Overview of the analytical processing of GPS data collected from collared wolves and moose in both northeastern Minnesota and the Voyageurs National Park ecosystem. (Wolf photographs provided by T. Gable and NPS staff) [Colour figure can be viewed at http://wileyonlinelibrary.com]

Our goal was to understand if and how wolf movement and resource use in two differing ecosystems alters the behavior of moose. Unfortunately, moose and wolf GPS data collected in the VNP ecosystem did not align temporally (wolves: December 2012–December 2015; moose: February 2010–February 2013). Moreover, although our GPS‐collared moose and wolves in NEMN had some spatial overlap (~58%), most of the overlap was among only a few wolf packs and several moose. Most wolf packs were located just to the south and east of the moose locations. To overcome the lack of temporal and spatial alignment, we followed a multistep process to analyze both moose and wolf data. We used models of wolf space use to generate predictions of how moose would behave in different habitats during different seasons and times of day. See Figure 2 for an overview of the process. First, (1) we determined times of day when individual wolves were moving the most (i.e., likely traveling or hunting); we accomplished this by modeling wolf movement rates as a function of time of day and habitat cover for each season. We later used these results to compare with intradaily moose movement patterns.

Next, (2) we created resource selection functions (RSFs) based on wolf pack habitat use for each study area and season. Using the RSF models, we predicted wolf habitat selection throughout each study ecosystem within areas where GPS‐collared moose were also found. While interannual variability in wolf space use has been documented (see Uboni, Vucetich, Stahler, & Smith, 2015), and in some cases, resource selection can be altered by local ecological conditions (see Kittle et al., 2017), we felt comfortable making the following simplifying assumptions when characterizing average wolf space‐use patterns: (a) resource selection did not change dramatically among years, (b) uncollared wolves behave similarly to collared wolves, and (c) the relationship between habitat variables and wolf space use remained consistent throughout each study area.

To capitalize on the high frequency of moose GPS locations, we used (3) first‐passage times (FPT = the time it takes an individual to move beyond a prespecified radius; Johnson, Wiens, Milne, & Crist, 1992) to capture fine‐scale (within patch) behavioral signals. Larger FPTs are associated with slower and more sinuous movements, reflective of rest and foraging behaviors, while moderate and small FPTs likely reflect movements between patches and movements to escape predators (Fauchald & Tveraa, 2003). FPT has been used to capture changes in movement behavior, including antipredator behavior and human avoidance, in elk (*Cervus elaphus*; Frair et al., 2005; Cleveland, Hebblewhite, Thompson, & Henderson, 2012), caribou (*Rangifer tarandus*; Le Corre, Dussault, & Côté, 2014), and roe deer (*Capreolus capreolus*; Le Corre et al., 2008).

At last, (4) we associated moose first‐passage times with mean wolf resource selection values within the same radius used to calculate the FPT. We modeled the relationship between log (moose) FPT and mean wolf RSF values to determine whether moose behavior changed from a more encamped behavior to more quick movements (i.e., less intensity of use/lower residency) when located in areas with predicted higher likelihood of wolf resource selection. We accounted for other temporal (Julian date, time of day, fix interval of moose GPS location [15 or 20 min]) and biological (sex of the moose) factors that may influence FPT in addition to wolf RSF predictions. We then compared the influence of hour on moose FPT times and compared these model estimates with hourly patterns of wolf movement rates modeled in component (1). We did not directly account for the influence of habitat and forage quality on moose FPT, but later we compare our findings with nearby studies of moose habitat selection and discuss how our findings relate to these studies.

#### 2.3.2 Wolf movement analysis

We removed individual GPS‐collared wolves with fewer than 50 locations in a given season. We included only consecutive locations 2–5 hr apart, and we subsampled locations <2 hr apart (spring = April–June; summer = July–October; winter = November–March). We calculated the distance (m) and fix interval (min) among sequential locations by individual wolf per season and created a movement rate associated with each set of GPS coordinates (distance [m]/fix interval duration [min]). We inspected histograms of seasonal wolf movement to make sure there were no outliers that were biologically infeasible (our estimated max. movement rate = 159 m per min).

We fit a generalized additive mixed model to the log of the wolf movement rate data by season and included a random intercept for each wolf ID using package “gamm4” (Wood & Scheipl, 2017) in program R (R Core Team 2016). This structure assumes wolves, regardless of pack structure, are independent and also that the effects of covariates are the same for all wolves. These assumptions are likely violated to some extent and therefore our standard errors for covariates that vary within an individual are likely to be optimistic (Schielzeth & Forstmeier, 2008). Nonetheless, this model provided a simple means to explore how movement patterns varied as a function of time of day, Julian date within the season, GPS fix interval durations (actual time between subsequent locations), and land cover types (agriculture [primarily hay/pasture in these areas], deciduous, conifer, and mixed forests, developed, herbaceous and woody wetlands, shrub/scrub, and open water) from the National Land Cover Database 2011 (Homer et al., 2015). We combined all land cover classifications for “herbaceous” and “hay/pasture” into herbaceous wetlands and agriculture, respectively, and we combined all levels of developed land cover classifications. We used a cyclic cubic regression spline (i.e., a penalized cubic regression spline where the ends must match) to model the relationship between hour of the day (*f* [time of day]) and log movement rate for each study area. We also used a smoothing spline to model the effect of Julian date. Wolf movement models for each study area and season were defined as log(movement rate)_*ij*_ ~ *f* (time of day, by = Study Area)_*ij*_ + *f* (month)_*ij*_ + fix interval duration_*ij*_ + *τ*_*j*_ + *ε*_*ij*_ for *j* = 1, 2, …, *n* individuals, where *τ* is a random intercept for each individual, (*ε*_*ij*_ ~ *N*(0, σ^2^_*ε*_) and *τ*_*j*_ ~*N*(0, σ^2^_*τ*_)). We checked the residuals and random intercept values for normality. We considered times where *f* (time of day) was >0 to be associated with relatively higher levels of activity (e.g., travel or hunting). We treated all covariates aside from the smoothing spline for time of day as nuisance variables.

#### 2.3.3 Wolf resource selection functions

We estimated wolf resource selection functions (RSFs) for each study area by season for a total of six RSFs (2 study areas × 3 seasons). RSFs compare the use of resources by the animal(s) relative to the total availability of the same resources within a defined area and provide an index of the relative likelihood of use given equal availability. We plotted and inspected individual wolf GPS locations at a monthly scale to identify whether any collared individuals were likely in the same pack as one or more other GPS‐collared individuals. We removed any individuals that were clearly dispersing (based on long‐distance travel and lack of settled range), and we pooled locations across individuals when their locations were in close spatial and temporal proximity. It is possible that not all GPS‐collared individuals were part of packs, but with the removal of dispersing individuals, included locations should accurately reflect resident wolf resource use. We considered individual packs/nondispersing individuals to be independent and fit individual RSF models to packs that had > 100 observations in a given season.

To delineate habitat availability, we created 95% kernel density estimates of home range for each pack by season using the ad hoc method for selecting a smoothing parameter with the adehabitatHR package (Calenge, 2006). We then removed any “wolf pack” GPS locations that fell outside the 95% seasonal home range and generated available points from within the home range using a random sample generated with the “spsample” function in the sp package (Pebesma & Bivand, 2005). The number of random points was based on the size of each wolf pack's seasonal home range size at a density of 1 random point per ha.

We used package “ResourceSelection” (Lele, Keim, & Solymos, 2017) in program R to model individual “wolf pack” RSFs, and we used the coefficients from each RSF model as “data” to create population‐level summaries (Murtaugh, 2007). Our models included the same nine land cover types from the National Land Cover Database that were used in the wolf movement analysis. We created indices for disturbance, primarily forestry cut blocks and fire in NEMN, based on a GIS layer that contained the spatial extent and period of disturbance (Garner, Nelson, Tavernia, Housman, & Perry, 2016). The indices assigned more recently disturbed areas a higher value (0–4, 0 = undisturbed 1990–2009; 1 = disturbed 1990–1994; 2 = disturbed 1995–1999; 3 = disturbed 2000–2004; 4 = disturbed 2005–2009). We calculated the distance (m) to the nearest water body larger than 5 ha, distance (m) to the nearest snowmobile trail (Minnesota Department of Natural Resources 2017a), distance (m) to the nearest state park trail/road (Minnesota Department of Natural Resources 2017b), and distance (m) to the nearest road (Minnesota Department of Transportation 2017) for each “wolf pack” location and corresponding available location. We included the shortest distance between each location and any of the three linear features (snowmobile, hiking trails, and roads) as a single variable. We included the slope (degrees) associated with each location based on digital elevation data collected with 30 m resolution by the United States Geological Survey.

We used the pack‐specific RSF models to generate season‐specific suitability values across each study area. For each pack, we calculated predicted RSF values by taking the product of the corresponding coefficients from the pack's RSF model and the spatial data (categorical land cover type, disturbance, distance to water body and linear feature, and slope) associated with the centroid of each 30 × 30 m raster cell, and then exponentiated the result. These values were then scaled by the sum of all cell values and multiplied by a constant (1,000,000). We then averaged RSF maps across packs. Occasionally, we needed to generate model predictions outside the range of covariate distances within a pack's home range (e.g., in some areas the distance to the nearest water body was greater than any observed distance). To avoid extrapolating and generating extremely large RSF values in these cases, we used the minimum distance of all observed maximum distance values within any pack's 95% home range when generating predicted RSF values in these cells. In VNP, some wolves had no locations in developed or agricultural land cover classifications, and we therefore could not estimate a coefficient for these two classes. As a result, we assigned the ~2% of raster cells containing these classifications an “NA” value for predictions from these packs in order to avoid influencing the mean value of the raster cell from pack's who had been located in them. In total, we developed six RSF prediction maps per study area (2 study areas × 3 seasons; Supporting Information Figure S1).

#### 2.3.4 Moose first‐passage time estimation

We removed GPS locations obtained from collared moose that had a large horizontal dilution of precision (HDOP > 15) and calculated movement rates to ensure they were biologically feasible (max. = 153 m per min). We split the GPS locations into seasons based on the same criteria used for the wolves (see above). Calculation of FPT requires data collected at regularly spaced time intervals with no missing GPS fixes. To meet this requirement, we imputed missing locations using linear interpolation whenever subsequent observations were less than 2 hr apart. We imputed data using the waddle package in R (Gurarie & Bracis, 2013). This process resulted in datasets in which 2.5% (NEMN) and 8.8% (VNP) of the locations were interpolated. http://FTPs can only be calculated if an animal's trajectory leaves the radius used for the FPT analysis. To minimize selection bias for short FPT times, we removed any movement path trajectories that did not include at least 10 consecutive GPS locations. We then removed any locations where we could not calculate a FPT value (i.e., locations where the moose never moved outside the radius, typically at the end of a trajectory; ~3.5% of data). Alternatively, intervals that were not long enough to observe a FPT could have been treated as right‐censored. We calculated FPT—that is, the amount of time it takes an individual animal to move beyond a given radius of space (Johnson et al., 1992)—for each location in the regularized and normalized dataset using package adehabitatLT (Calenge, 2006). Because we were interested in determining the relationship between wolf RSF values in time and space with corresponding moose behavior, we chose a relatively small radius of 45 m for all 15 min interval data (VNP only), and 60 m for 20 min interval data (VNP and NEMN to make the data collected at different intervals more comparable). We chose the radius sizes to ensure they were larger than the average GPS error of the collars to estimate resting behavior properly.

We overlaid the buffered areas used to calculate each moose FPT on the appropriate wolf RSF map (i.e., matching the location to the appropriate moose study area and season). We then extracted the mean wolf RSF value within these areas. We averaged and plotted each individual moose‐year FPT by study area and season and calculated 95% confidence intervals of the average FPTs for each of these strata to look for temporal patterns that should be accounted for when modeling moose FPT (Supporting Information Figure S2).

#### 2.3.5 Modeling moose first‐passage time

We fit linear mixed effect models with package “lme4” (Bates, Mächler, Bolker, & Walker, 2014) in program R to model data from each study area and season (spring, summer, winter) by regressing the log of moose FPT (hr) as a function of: (a) predicted mean wolf RSF, centered and scaled, (b) hour of the day, (c) Julian date, (d) sex of the moose, and (e) a factor for fix interval of the moose locations (either 15 or 20 min) in the VNP models only (all NEMN were 20 min intervals). We allowed for nonlinear relationships using regression splines with (2, 5, and 2 *df*) for wolf RSF, hour of day, and Julian date, respectively. We included a random intercept for moose‐year and the *f* (rsf) was also allowed to vary by animal with the expectation that annual changes in weather, reproductive status and health would greatly alter moose behavior from 1 year to the next. We used the package “lmerTest” (Kuznetsova, Brockhoff, & Christensen, 2017) to obtain *p*‐values for each coefficient using Satterthwaite's method, and we used the “effects” package (Fox, 2003) to generate model‐based predictions of moose FPT with associated 95% confidence intervals to visualize effect sizes of covariates of particular interest, especially those modeled using splines. We reported and plotted the mean predicted FPT value (±95% confidence interval) at the 0.5th and 99.5th quantile of the centered and scaled wolf RSF value and when the centered and scaled wolf RSF = 0 (i.e., at the mean wolf RSF) in each study area and season. When making predictions from the VNP model, we assumed a 20‐min fix interval to make predictions more comparable to NEMN.

#### 2.3.6 Post hoc sensitivity analyses

Analyses that utilize a multistep modeling process with numerous biological and methodological assumptions may introduce uncertainty in the first steps that could impact the conclusions. We conducted a sensitivity analysis to determine whether we could still detect the same signal in the relationship between wolf RSF and moose FPT if we used ±1 *SE* of our predicted wolf RSF values in our current model instead of the mean wolf RSF values found within each FPT radius. We also tested the sensitivity of this relationship by removing any moose FPT estimates associated with predicted wolf RSF values influenced by our modeling decision of setting a maximum distance for prediction based on observed GPS locations within wolf home ranges.

## 3 RESULTS

### 3.1 Wolf movement

To model wolf movement rates, we used data from three wolves in spring (obs. = 859), 10 during summer (obs. = 4,223), and five during winter (obs. = 3,468) from NEMN. In VNP, we used data from four wolves during spring (obs. = 1,016), 11 during summer (obs. = 7,202), and 12 in winter (obs. = 5,519). Average wolf movement rates in NEMN were similar across the 3 seasons (spring: $\overset{¯}{X}$ = 5.7 m per min, 95% CI = 5.2–6.1 m per min; summer: $\overset{¯}{X}$ = 5.8, 95% CI = 5.7–6.0; winter: $\overset{¯}{X}$ = 6.1, 95% CI = 5.9–6.3). VNP wolf average movement rates were similar in summer and winter (summer: ¯X= 6.1, 95% CI = 5.9–6.3; winter: ¯X= 6.2, 95% CI = 5.8–6.4), but slower in spring (spring: ¯X= 4.5, 95% CI = 3.9–5.0).

**Figure 3:**
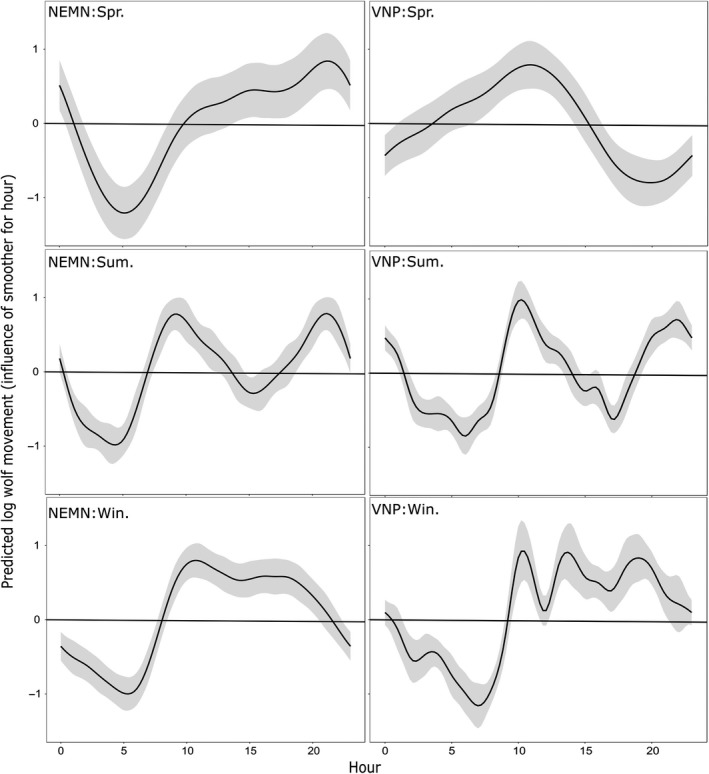
Influence of hour of the day on predicted log wolf movement rates (mean and 95% pointwise confidence intervals) in northeastern (NEMN) Minnesota and the Voyageurs National Park (VNP) ecosystem. Temporal trends within a day were modeled using cyclical smoothing splines in seasonal generalized additive mixed models fit to log movement rates of all wolves with random intercepts based on wolf ID. Models also included covariates for land cover type, GPS fix interval durations, and a smoother for Julian date. Seasons were delineated as spring = April–June; summer = July–October; and winter = November–March

Intradaily movement patterns were similar in NEMN and VNP during summer and winter, but not during spring (Figure 3), after accounting for the influences of landcover type, Julian date and fix interval. During summer, wolf movements were fastest starting in the crepuscular times of day (~7:00–11:00) and (~18:00–24:00). Wolves were mostly diurnal during winter (~09:00–19:00). In spring, VNP wolf activity was more diurnal (~06:00–14:00), whereas NEMN wolves were active later ~11:00–24:00. We caution that this discrepancy during spring may be due to the relatively few wolf packs and GPS locations collected during this time of year.

### 3.2 Wolf resource selection

During all seasons, wolves in both study areas (spring: NEMN, *n* = 4; VNP, *n* = 6; summer: NEMN, *n* = 10; VNP, *n* = 17; winter: NEMN, *n* = 5; VNP, *n* = 16) selected for mixed forest cover (i.e., most coefficients for habitat categories were negative relative to mixed forest baseline; Table 1). Wolves avoided agricultural, developed, open water/ice, and scrub/shrub land cover (Table 1). In spring, NEMN wolves avoided wetlands, while VNP wolves selected for them and avoided conifer cover. VNP avoided areas with shrub/scrub during the summer and winter.

**Table 1:** Mean coefficient values and 95% confidence intervals of wolf resource selection functions fit to individual packs or individuals in northeastern Minnesota (NEMN) and Voyageurs National Park (VNP) ecosystem

Coefficient	Spring: Mean (±95% CI)		Summer: Mean (±95% CI)		Winter: Mean (±95% CI)	
	NEMN	VNP	NEMN	VNP	NEMN	VNP
Deciduous forest	−0.25 (−0.36, −0.17)	−0.06 (−0.23,0.12)	0.10 (−0.22,0.35)	0.52 (−0.20,1.99)	−0.09 (−0.53,0.30)	−0.09 (−0.26,0.11)
Conifer forest	−0.48 (−1.11, −0.14)	0.07 (−0.08,0.27)	−0.17 (−0.42,0.07)	−6.43 (−13.6, −0.40)	−0.06 (−0.51,0.42)	−1.11 (−5.30,0.02)
Shrub/scrub	−0.33 (−0.93,0.06)	−0.93 (−4.14, −0.01)	0.06 (−0.52,0.47)	−2.96 (−12.96,0.64)	−0.26 (−0.66,0.03)	−1.02 (−4.56, −0.02)
Woody wetland	−0.79 (−1.22, −0.43)	0.87 (0.54,1.15)	−0.11 (−0.36,0.11)	0.19 (−0.31,0.47)	−0.20 (−0.93,0.06)	−0.01 (−0.35,0.27)
Herbaceous wetland	−0.25 (−0.49, −0.04)	0.14 (0.02,0.24)	0.45 (−0.08,0.91)	−2.77 (−13.66,1.25)	−0.12 (−0.39,0.07)	0.12 (0.02,0.27)
Open water/ice	−6.23 (−17.35, −0.61)	−4.65 (−8.18, −2.34)	−1.81 (−2.96, −1.12)	−4.49 (−11.40, −1.94)	−2.47 (−5.63, −1.32)	−2.47 (−5.80, −1.38)
Developed	−6.82 (−17.01, −1.63)	−5.08 (−9.53, −1.56)	−0.78 (−1.40, −0.27)	−4.34 (−11.40, −0.73)	−1.00 (−1.37, −0.79)	−7.48 (−10.58, −4.15)
Agricultural	−9.18 (−16.74, −3.64)	0.20 (0.02,0.39)	−9.12 (−10.25, −6.57)	−10.08 (−18.11, −4.17)	−9.42 (−12.81, −3.5)	0.02 (−0.45,0.22)
Disturbance level (0–4)	0.07 (−0.05,0.16)	0.00 (−0.01,0.01)	0.12 (0.06,0.17)	−2.75 (−9.75, −0.30)	0.08 (−0.03,0.17)	0.01 (0.00,0.02)
Slope	−0.05 (−0.07, −0.04)	−10.11 (−12.58, −5.96)	−0.01 (−0.03,0.00)	0.00 (−0.04,0.04)	0.00 (−0.01,0.02)	−7.98 (−10.6, −4.26)
Distance (km) to linear feature	−0.42 (−0.64, −0.26)	−0.07 (−0.18,0.09)	−0.49 (−0.87, −0.25)	0.05 (−0.24,0.23)	−0.31 (−0.56, −0.10)	−0.12 (−0.27,0.00)
Distance (km) to water body	−0.09 (−0.26,0.00)	−0.47 (−1.01, −0.04)	0.02 (−0.10,0.14)	−0.61 (−2.80,1.54)	−0.16 (−0.49,0.01)	−0.31 (−0.73, −0.01)

Wolves in both study areas selected for disturbed sites during summer and winter (Table 1). Wolves in NEMN consistently selected for areas closer to linear features, while VNP wolves were more attracted to areas near water bodies. NEMN wolves avoided steeper slopes in spring.

### 3.3 Moose first‐passage time

We calculated FPTs for 22 moose (moose‐years = 44; 769,245 obs.) in NEMN and 19 moose (moose‐years = 43; 619,391 obs.) in VNP. Among the seasonal NEMN models, winter included the largest number of moose‐years (*n* = 41; obs. = 278,988), followed by spring (*n* = 33; obs. = 202,763) and summer (*n* = 30; obs = 235,152). VNP seasonal models consisted of 39 moose‐years (obs. = 188,113) in the winter model, 33 moose‐years (obs. = 154,044) in spring, and 22 moose‐years (obs. = 203,897) in the summer.

**Figure 4:**
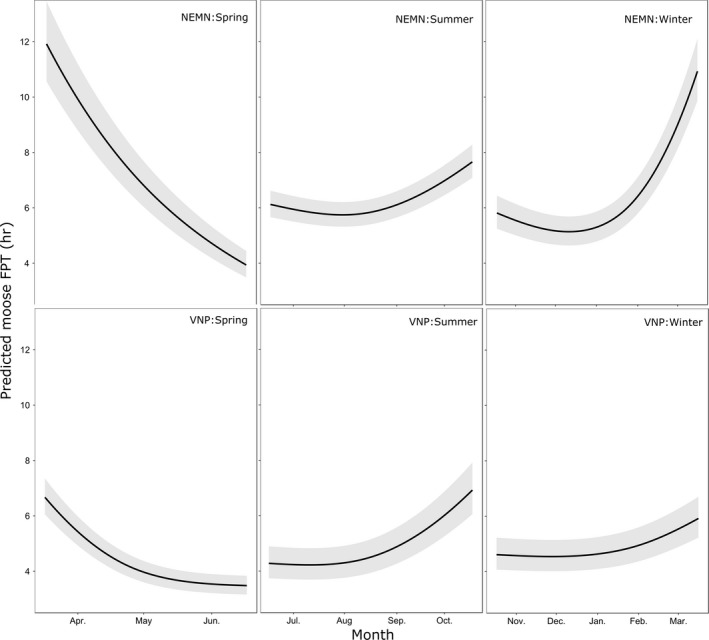
Influence of Julian date on predicted moose first‐passage time (mean and 95% pointwise confidence intervals) for GPS‐collared moose in northeastern Minnesota (NEMN) and the Voyageurs National Park (VNP) ecosystem using seasonal models (spring = April–June; summer = July–October; and winter = November–March). Other continuous predictors were set to their mean values, and categorical predictors were set to their mode except for fix rate which was set at 20 min for easier comparisons between the two study sites

In both NEMN and VNP, estimated FPTs varied throughout the year, with the highest FPTs occurring during March–April and October (Figure 4). Moose FPTs were the shortest during the months of June and July. Fix intervals of 20 min resulted in longer FPTs relative to GPS locations collected every 15 min in VNP despite the smaller buffer area used for the FPT analysis of 15 min data (Supporting Information Tables S1 and S3). During spring and summer, male moose had lower FPTs relative to females (Supporting Information Tables S1–S3).

### 3.4 Moose FPT's relationship to wolf spatial and temporal patterns

**Figure 5:**
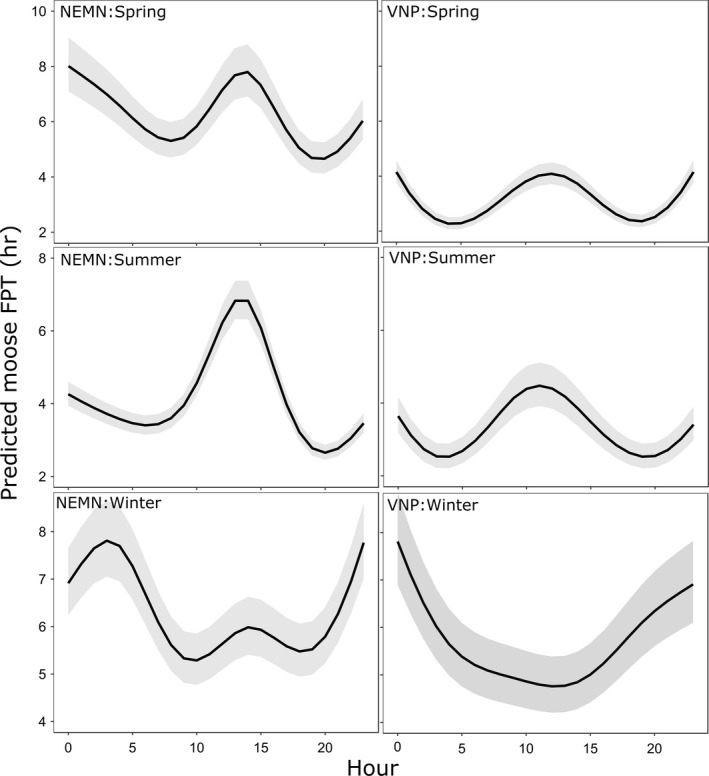
Seasonal influence of hour on predicted moose first‐passage time (mean and 95% pointwise confidence intervals) for GPS‐collared moose in northeastern Minnesota (NEMN) and the Voyageurs National Park (VNP) ecosystem. Seasons were defined as: spring = April–June; summer = July–October; and winter = November–March. Other continuous predictors were set to their mean values, and categorical predictors were set to their mode except for fix rate which was set at 20 min for easier comparisons between the two study sites

Moose exhibited strong intradaily patterns of FPT that varied by season, but were very similar among study areas (Figure 5). During winter, moose were primarily diurnal (smallest FPTs; associated with faster movements) which coincides with the timing of the highest amounts of wolf activity (Figures 3 and 5). Moose movements also aligned with wolf movement periods during the summer when both species were primarily active in crepuscular and nighttime hours. First‐passage times during spring were also smallest during crepuscular and nighttime hours, which aligns with the fastest movement period of NEMN, but not VNP wolves (Figures 3 and 5).

**Figure 6:**
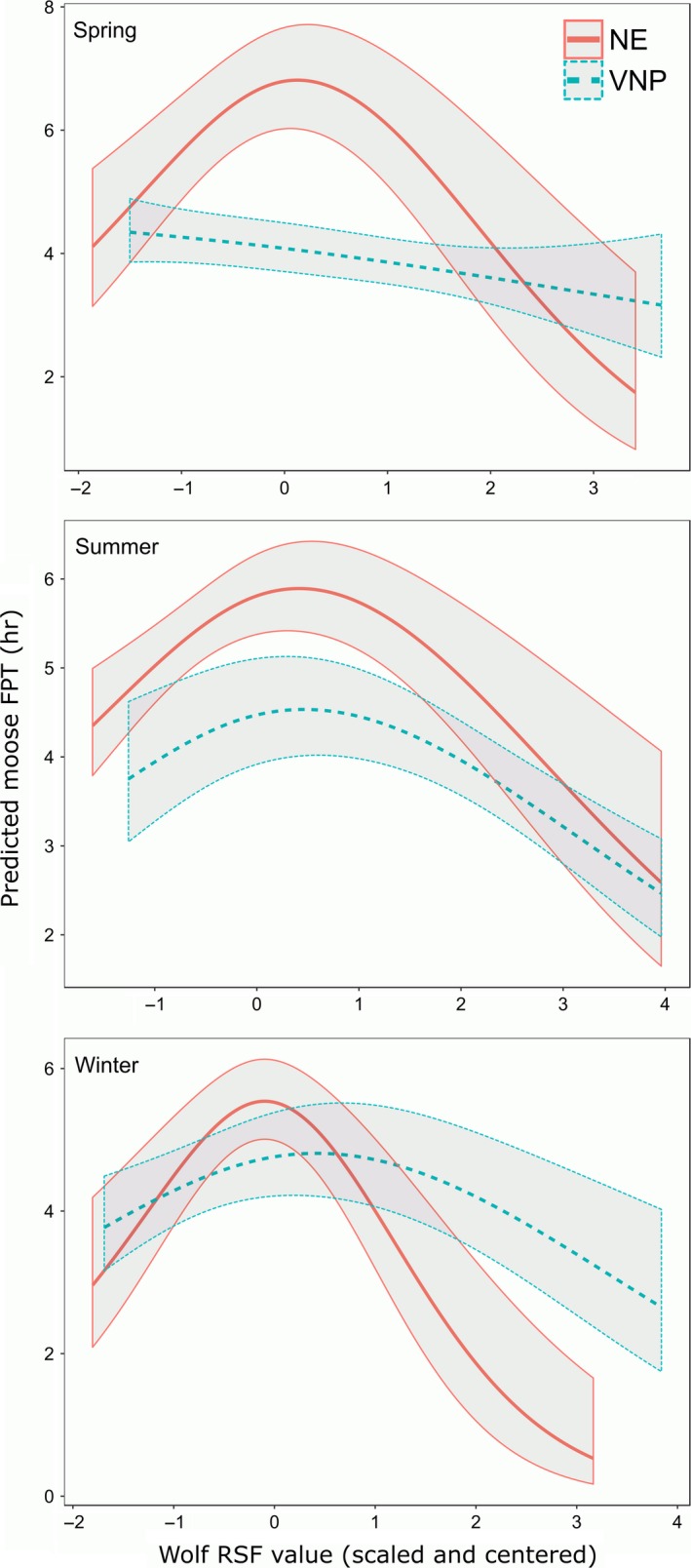
Relationship between wolf RSF (scaled and centered) and moose first‐passage time (FPT; mean and 95% pointwise confidence intervals) by study area and season. GPS‐collared moose and wolves were located in the study areas of northeastern Minnesota (NEMN) and the Voyageurs National Park (VNP) ecosystem. Seasons were defined as: spring = April–June; summer = July–October; and winter = November–March. Moose FPT areas were overlaid onto the corresponding wolf resource selection prediction maps that corresponded to study area and season. We used linear mixed models to assess the influence of predicted wolf RSF values on moose FPT, and we made predictions across the 99% quantile range of observed wolf RSF values in a given study area and season. Other continuous predictors were set to their mean values and categorical predictors were set to their mode except for fix rate which was set at 20 min for easier comparisons between the study sites [Colour figure can be viewed at http://wileyonlinelibrary.com]

Moose reduced their intensity of use in areas where we predicted a higher likelihood of wolf resource selection (Figure 6; Supporting Information Tables S1–S3). The relationship between wolf RSF and moose FPT was nonlinear in most seasons with peak FPT values occurring when centered and scaled wolf RSF values were 0 (i.e., for mean levels of the wolf RSF). On average across seasons and study areas, moose reduced FPT by 45% when comparing areas with the lowest to those with the highest relative likelihood of wolf resource selection; representing a reduction in time equivalent to 1 hr and 44 min (Table 2; Figure 6). The average reduction increased to 55% (time equivalent = 3 hr and 3 min [3.05 hr]) when comparing the lowest to the mean values of predicted wolf resource use. On average across seasons, moose FPT values in NEMN were more strongly negatively correlated than VNP moose with wolf RSF values (Table 2; Figure 6). In NEMN, moose FPT was reduced most during winter based on increased predicted likelihood of wolf resource selection, followed by spring (Table 2; Figure 6). In contrast, we estimated the largest reductions in VNP moose FPT in relation to increasing wolf RSF values during summer and winter (Table 2; Figure 6). In addition to larger percentage changes among NEMN moose relative to VNP, it should be noted that NEMN has much larger FPT values in general, therefore the effect of increased wolf RSF values on predicted time spent in an area is much greater in terms of moose movement behavior overall (Table 2; Figures 4, 5, 6). Relationships between RSF values and FPTs were robust to uncertainty in the estimated RSF values (Supporting Information Figure S3) and when removing moose FPTs associated with locations outside of the observed maximum distances of wolf GPS locations with distance‐related values (linear features and water bodies).

**Table 2:** Seasonal predicted effects of wolf RSF on moose first‐passage time (mean and 95% pointwise confidence intervals) for GPS‐collared moose in northeastern Minnesota (NEMN) and the Voyageurs National Park (VNP) ecosystem by study area and season based on linear mixed models. We report on the values associated with the 0.5th quantile, where the predicted wolf RSF = 0 (mean value), and the 99.5th quantile of observed wolf resource selection values along with the percentage change between the 99.5th quantile and each value. The effect of wolf RSF was modeled using regression splines with two degrees of freedom

Study area	Wolf RSF value	Spring		Summer		Winter	
		Mean (95% confidence interval)	Mean FPT change (%)	Mean (95% confidence interval)	Mean FPT change (%)	Mean (95% confidence interval)	Mean FPT change (%)
NE	0.5 percentile	4.11 (3.14, 5.38)	57.6	4.35 (3.79, 4.99)	40.5	2.96 (2.09, 4.19)	82.1
	Zero	6.79 (6.02, 7.66)	74.4	5.81 (5.38, 6.28)	55.5	5.52 (4.99, 6.12)	90.4
	99.5 percentile	1.74 (0.82, 3.70)	—	2.59 (1.65, 4.06)	—	0.53 (0.17, 1.66)	—
VNP	0.5 percentile	4.34 (3.86, 4.89)	27.2	3.75 (3.05, 4.62)	34.3	3.77 (3.16, 4.49)	29.7
	Zero	4.08 (3.71, 4.50)	22.6	4.47 (3.92, 5.11)	44.9	4.76 (4.21, 5.38)	44.3
	99.5 percentile	3.16 (2.32, 4.32)	—	2.47 (1.98, 3.08)	—	2.65 (1.75, 4.02)	—

## 4 DISCUSSION

It is well established that predator presence influences prey in many nonlethal ways that modify where and how prey spend their time; when prey forage in risky areas, they use behavioral adaptations, such as increased vigilance and reduced time allocation (Brown & Kotler, 2004; Brown, Laundré, & Gurung, 1999; McArthur, Banks, Boonstra, & Forbey, 2014). Our study quantified how the predicted likelihood of predator presence on the landscape was related to the movement rates of prey. We found moose moved faster when traversing areas with higher predicted likelihoods of wolf resource use, and during times of day when wolves were more active. We detected these changes by examining alterations in moose movement or intensity of use in comparison with predicted resource selection and periods of activity by wolves even without data collected from animals that interacted regularly or were monitored concurrently. We tested the strength of the relationship between moose behavior and predicted wolf resource selection in two study systems in northern Minnesota. Our results supported the hypothesis that moose movement rates were positively correlated with higher predicted resource use. A dietary analysis of wolves in both systems by Chenaux‐Ibrahim (2015) revealed wolf diets in VNP contained far less moose (only ~2% of wolf scats; *n* = 235) compared to NEMN, and moose were only present in scat during spring and winter. Although abundance of moose in VNP is relatively low, adult survival is high (91% annual survival, estimated from 2010 to 2016; Windels & Olson, 2016). In contrast, moose were found in ~31% of wolf scats (*n* = 243) in NEMN throughout the year and adult survival is lower (Carstensen et al., 2015; Lenarz, Nelson, Schrage, & Edwards, 2009). Thus, moose in NEMN reduced first‐passage time in areas that wolves frequent throughout the year and the average response was larger in NEMN compared to VNP.

Prey populations that must engage in chronic antipredator behavior, such as vigilance, can suffer behaviorally mediated deleterious consequences as a result of forgone foraging opportunities (Creel et al., 2007). The spatial dynamics between predator and prey can be difficult to disentangle, especially when prey select for areas that predators avoid and vice versa (Sih, 2005). FPTs have been shown to be useful for evaluating how ungulate prey respond to the likelihood of predator encounter (see Cleveland et al., 2012; Frair et al., 2005; Le Corre et al., 2008, 2014), and they have provided insights beyond considering resource selection alone. The reduction we observed in moose FPT of over 40% (equivalently a reduction in nearly 2 hr) between areas of low to high predicted likelihood of wolf resource use in both northern MN ecosystems was surprisingly large and may make it difficult for moose to balance activity budgets. This consistent reduction in the intensity of use in areas with higher likelihoods of wolf resource use likely represents prey adapting to predator. By contrast, shorter FPT values exhibited in areas with low wolf RSF values may be suggestive of either predator adapting to prey or a common avoidance of certain habitat characteristics. Other studies have quantified large costs associated with antipredator behavior as well. Christianson and Creel (2010) found that elk avoided areas used by wolves resulting in a dietary deficiency equal to 27% of maintenance requirements. Using a similar approach to our analysis, Frair et al. (2005) combined FPT of a prey species, elk, with RSF of wolves and reported longer elk FPTs in areas least likely to be used by wolves, and shorter FPTs in areas more likely to be frequented.

It is important to consider not only the magnitude of antipredator behavioral changes, but also to understand how these changes might influence access to forage availability and important habitats. A fine‐scale analysis of caribou (*Rangifer tarandus*) and moose movements in relation to the passage of wolves by Latombe et al. (2014) revealed that after some encounters both movement and habitat selection changed for several days. Most important, the ungulates reduced their use of preferred habitats for foraging in favor of safer ones. Our study, similar to a study by Kittle et al. (2017) in Ontario, found that wolves selected for several land cover types and landscape features that are also of critical importance to moose. NEMN wolves selected for close proximity to linear features which moose generally tend to avoid except when seeking vegetation enriched with sodium from road maintenance (Laurian et al., 2008). Whereas roads and other linear features are rare in the VNP ecosystem where moose were collared, the NEMN study area contains a much higher density of linear features that wolves utilize for faster movement when hunting (Dickie, Serrouya, McNay, & Boutin, 2017; James & Stuart‐Smith, 2000; McKenzie, Merrill, Spiteri, & Lewis, 2012; Whittington, St. Clair, Cassady, & Mercer, 2004; Whittington, St. Clair, & Mercer, 2005). Wolves in VNP were attracted to the shores of large water bodies (not NEMN wolves) which supports the findings of several previous studies that found selection for lakeshores throughout the year (Montgomery, Vucetich, Peterson, Roloff, & Millenbah, 2013; Peterson, 1975), and our findings also indicate support for studies showing wolf selection for disturbed areas (Ballard, Krausman, Boe, Cunningham, & Whitlaw, 2000; Fisher & Wilkinson, 2005; Kuzyk, Kneteman, & Schmiegelow, 2004). Lakeshores are important for moose when foraging for aquatic vegetation and avoiding biting insects (Morris, 2014), and disturbed areas offer moose nutritious young vegetation (Telfer, 1984). Wolves primarily selected for mixed deciduous–conifer forests and woody wetlands (VNP wolves in summer), which Street et al. (2016) found were strongly selected for by moose in NEMN. Wolves strongly avoided agriculture and developed land cover classes, which moose also avoid except in some ecosystems where moose keep close proximity to human‐influenced areas as an antipredator shield when calving (Berger, 2007). The common avoidance of these habitat classes by both wolves and moose in NEMN, where developed and agricultural areas (agriculture was primarily hay/pasture) are more common than VNP, may explain the positive component of the nonlinear relationship between moose FPT and wolf RSF at the areas with the lowest predicted resource selection by wolves (i.e., low moose FPT corresponds with low wolf RSF values).

It is often difficult to establish causation in many predator–prey systems, and in the context of our methods, areas where moose choose to forage should result in larger FPT estimates, yet if wolves are consistently seeking areas where moose spend the most time, FPT estimates would then be reduced because of predator avoidance. The same difficulty for discerning causation occurs when considering the high degree of temporal overlap in the times of day that moose and wolves are most active. However, the consistent correlative relationship between areas of predicted wolf resource use and moose FPT does suggest that moose may be altering their movement through areas at least in part due to a perceived heightened risk of a predator encounter or attack success (Hebblewhite et al., 2005). If antipredator behavior is the cause of increased movement rates, it can reduce time spent on critical activities (that would increase FPT), such as foraging, breeding and finding bed sites and thermal shelter (see McCann, Moen, & Harris, 2013; Renecker & Hudson, 1986, 1989). Disruptions to activity budgets can be especially detrimental to populations of moose along their southern geographic extent because they are prone to heat stress, and many southern populations have exhibited signs of poorer health and/or reductions in population size (Dou et al., 2013; Grøtan et al., 2009; Monteith et al., 2015; Ruprecht et al., 2016); including within Minnesota (ArchMiller et al., 2018; Lenarz, Fieberg, Schrage, & Edwards, 2010; Lenarz et al., 2009; Murray et al., 2006). Numerous studies show the links between elevated ambient temperatures and behavioral and habitat selection changes in moose (Ditmer et al., 2017; McCann et al., 2013, 2016; Street et al., 2016). Moose in both study systems exhibited consistent and strong intradaily movement patterns that align with ambient temperatures (less movement mid‐day during spring and summer, more movement mid‐day during winter).

Seasonal changes in the association between moose FPT and wolf RSF may reflect differing prey selection by wolves in NEMN and VNP. We hypothesized, but did not find support, that moose would respond the most to areas with higher predicted wolf selection during spring because moose give birth in May, and a sizable percentage of young calves are killed by wolves (Severud et al., 2015). Instead, we found the largest effects in NEMN during winter and similar results among all seasons for VNP moose. The abundance and availability of alternative prey items, especially white‐tailed deer, may be playing a more critical role in shaping the seasonal relationships between moose and wolves in these different ecosystems. In VNP, both abundant deer (2–4 deer/km^2^; Gable, Windels, & Bruggink, 2017; Gable, Windels, & Olson, 2017) and beaver populations (~5.0 beaver/km^2^; Johnston & Windels, 2015) appear to provide plentiful alternative prey for wolves (Chenaux‐Ibrahim, 2015; Gable et al., 2016; Gogan et al., 2004). In NEMN, beaver are much less common, and the deer population is semimigratory, and thus, their availability is greatly reduced during winter and part of spring (Fieberg et al., 2008; Nelson, 1995). As a result, wolves frequently target moose that are in their weakest physical state during the winter (Schwartz & Renecker, 2007). A study by Barber‐Meyer and Mech (2016) examined changes in population abundance and wolf dietary estimates between 2002 to 2011 and found support for apparent competition between deer and moose in the NEMN study area. During this period, wolf populations increased (~doubled in the study area) despite the decline of moose populations. Barber‐Meyer and Mech (2016) concluded that wolf populations did not suffer declines when moose populations declined because they were able to switch their primary prey to deer, yet the wolves continued to have a major influence on the moose population through continued elevated levels of calf predation (see also Mech et al., 2018).

Studies which use GPS‐tagged individuals to identify animal behavior typically assume that a subset of collared individuals is representative of all individuals in a population and that collecting data over the course of multiple years captures enough variation to reasonably represent the population. However, even when many individuals and interactions are captured with GPS tags over several years, it can be especially difficult to quantify the relationships between predator and prey or to isolate or infer a behavioral response (Eriksen et al., 2011). Well‐designed studies can still suffer from limited spatial overlap among tagged individuals based on avoidance, seasonal migrations or insufficient sample size, and temporal misalignment that can be caused by logistical or technical problems in animal capture or GPS unit deployment. In addition, many analytical methods require researchers to subset GPS locations to include only those locations that overlap temporally and spatially at some threshold distance and time intervals between predator and prey.

Our analysis, which integrated the likelihood of wolf resource use (using resource selection functions) with moose behavior (via first‐passage time), overcame spatial misalignment of GPS‐collared individuals in one ecosystem and temporal misalignment in another and found strong correlation in the movement behavior of a prey species to varying degrees in two ecosystems. Although caution needs to be taken when extrapolating from RSFs (Northrup, Hooten, Anderson, & Wittemyer, 2013), our predictions only expanded marginally outside of our study areas, and we took care to limit the prediction model inputs to values that occurred within the home range of each wolf pack (as well as conducting multiple sensitivity analyses). However, we acknowledge that modeling such a dynamic system requires numerous assumptions and likely there are far more factors influencing this relationship between predator and prey resulting in unaccounted uncertainty in our models. We also note that it is difficult to establish cause and effect. It is also possible that wolf resource selection and activity periods may be driven by wolves attempting to increase the likelihood of encountering a moose rather than the other way around. Regardless of the directionality, understanding how moose behavior is related to predator presence is important when trying to understand the causes and contributing factors to ungulate population declines. Our findings help to further identify how the influence of predators might strain the activity budgets of moose and result in foraging or other opportunity costs. Future research that measures the stress levels in the declining NEMN moose population relative to other healthier populations could quantify the effects wolves have on the physiology of moose. In addition, as the technology and battery life of GPS collars improve, multispecies geofencing and video‐enabled collars will further our understanding of predator–prey dynamics by connecting animal responses to GPS movement characteristics.

## CONFLICT OF INTEREST

None declared.

## AUTHOR CONTRIBUTIONS

TRH, RAM, and SKW conceptualized the study. RAM and SKW collected the data. MAD designed the analysis with input from JRF and SPP. MAD wrote the initial draft of the manuscript. JRF, RAM, SKW, SPP, and TRH provided input and edits.

## DATA ACCESSIBILITY

Final datasets used for wolf movement, wolf resource selection, and moose first‐passage time models are available at the Dryad Digital Repository: https://doi.org/10.5061/dryad.k0503c7
