# Supplementary material for: Moose movement rates are altered by wolf presence in two ecosystems
Source: Ecol Evol. 2018 Aug 19;8(17):9017–33. doi: 10.1002/ece3.4402 (PMC6157672; doi:10.1002/ece3.4402)

**Table S1.)** Regression parameter estimates, standard errors, t-statistics and p-values for the covariates included in the spring linear mixed effects models assessing factors influencing moose first-passage time of GPS-collared moose in northeastern Minnesota (NEMN) and the Voyageurs National Park ecosystem (VNP). Spring included the months of April through June. The fix interval covariate was only included in the VNP model because it was the only study area with moose that had both 15 and 20 min fix intervals.

| **Study Area** | **Covariates*** | **Beta** | **SE(Beta)** | ***t*** | ***P*** |
| --- | --- | --- | --- | --- | --- |
| NEMN | Intercept | 2.05 | 0.18 | 11.24 | < 0.001 |
|  | Hour S1 | -0.63 | 0.01 | -45.67 | < 0.001 |
|  | Hour S2 | 0.33 | 0.02 | 19.85 | < 0.001 |
|  | Hour S3 | -0.74 | 0.01 | -55.02 | < 0.001 |
|  | Hour S4 | -0.59 | 0.02 | -24.00 | < 0.001 |
|  | Hour S5 | -0.18 | 0.01 | -16.46 | < 0.001 |
|  | Julian Date S1 | -1.32 | 0.02 | -78.87 | < 0.001 |
|  | Julian Date S2 | -0.85 | 0.01 | -93.49 | < 0.001 |
|  | Sex: male | -0.35 | 0.13 | -2.65 | 0.013 |
|  | Wolf RSF S1 | -0.11 | 0.43 | -0.27 | 0.79 |
|  | Wolf RSF S2 | -3.50 | 0.93 | -3.77 | < 0.001 |
| VNP | Intercept | 1.59 | 0.11 | 14.29 | < 0.001 |
|  | Hour S1 | -0.05 | 0.01 | -3.34 | < 0.001 |
|  | Hour S2 | 0.09 | 0.02 | 5.30 | < 0.001 |
|  | Hour S3 | -0.64 | 0.01 | -48.68 | < 0.001 |
|  | Hour S4 | -1.02 | 0.02 | -42.57 | < 0.001 |
|  | Hour S5 | 0.52 | 0.01 | 47.98 | < 0.001 |
|  | Julian Date S1 | -1.04 | 0.02 | -61.90 | < 0.001 |
|  | Julian Date S2 | -0.39 | 0.01 | -41.46 | < 0.001 |
|  | Sex: male | -0.23 | 0.11 | -2.06 | 0.048 |
|  | Wolf RSF S1 | -0.46 | 0.15 | -3.11 | 0.004 |
|  | Wolf RSF S2 | -0.57 | 0.45 | -1.25 | 0.22 |
|  | Fix interval: 20 min | 0.65 | 0.11 | 5.84 | < 0.001 |

*”S” stands for spline

**Table S2.)** Regression parameter estimates, standard errors, t-statistics and p-values for the covariates included in the summer linear mixed effects models assessing factors influencing moose first-passage time of GPS-collared moose in northeastern Minnesota (NEMN) and the Voyageurs National Park ecosystem (VNP). Summer included the months of July through October. The fix interval covariate was only included in the VNP model because it was the only study area with moose that had both 15 and 20 min fix intervals.

| **Study Area** | **Covariates*** | **Beta** | **SE(Beta)** | ***t*** | ***P*** |
| --- | --- | --- | --- | --- | --- |
| NEMN | Intercept | 1.10 | 0.11 | 9.91 | < 0.001 |
|  | Hour S1 | -0.32 | 0.01 | -27.76 | < 0.001 |
|  | Hour S2 | 1.01 | 0.01 | 73.30 | < 0.001 |
|  | Hour S3 | -0.73 | 0.01 | -65.77 | < 0.001 |
|  | Hour S4 | -0.55 | 0.02 | -27.66 | < 0.001 |
|  | Hour S5 | -0.09 | 0.01 | -9.91 | < 0.001 |
|  | Julian Date S1 | 0.01 | 0.01 | 0.98 | 0.33 |
|  | Julian Date S2 | 0.28 | 0.01 | 34.98 | < 0.001 |
|  | Sex: male | -0.21 | 0.07 | -3.16 | 0.004 |
|  | Wolf RSF S1 | 0.10 | 0.31 | 0.32 | 0.75 |
|  | Wolf RSF S2 | -2.07 | 0.59 | -3.54 | 0.002 |
| VNP | Intercept | 0.46 | 0.24 | 1.93 | 0.062 |
|  | Hour S1 | 0.24 | 0.01 | 22.89 | < 0.001 |
|  | Hour S2 | 0.22 | 0.01 | 15.66 | < 0.001 |
|  | Hour S3 | -0.50 | 0.01 | -44.67 | < 0.001 |
|  | Hour S4 | -0.72 | 0.02 | -38.05 | < 0.001 |
|  | Hour S5 | 0.33 | 0.01 | 38.94 | < 0.001 |
|  | Julian Date S1 | 0.27 | 0.01 | 20.53 | < 0.001 |
|  | Julian Date S2 | 0.50 | 0.01 | 68.01 | < 0.001 |
|  | Sex: male | -0.48 | 0.11 | -4.35 | < 0.001 |
|  | Wolf RSF S1 | 0.30 | 0.22 | 1.35 | 0.19 |
|  | Wolf RSF S2 | -1.21 | 0.22 | -5.38 | < 0.001 |
|  | Fix interval: 20 min | 0.56 | 0.19 | 2.99 | 0.007 |

*”S” stands for spline

**Table S3.)** Regression parameter estimates, standard errors, t-statistics and p-values for the covariates included in the winter linear mixed effects models assessing factors influencing moose first-passage time of GPS-collared moose in northeastern Minnesota (NEMN) and the Voyageurs National Park ecosystem (VNP). Winter included the months of November through March. The fix interval covariate was only included in the VNP model because it was the only study area with moose that had both 15 and 20 min fix intervals.

| **Study Area** | **Covariates*** | **Beta** | **SE(Beta)** | ***t*** | ***p*** |
| --- | --- | --- | --- | --- | --- |
| NE | Intercept | 1.24 | 0.21 | 5.86 | < 0.001 |
|  | Hour S1 | -0.47 | 0.01 | -53.41 | < 0.001 |
|  | Hour S2 | 0.00 | 0.01 | 0.20 | 0.84 |
|  | Hour S3 | -0.44 | 0.01 | -52.61 | < 0.001 |
|  | Hour S4 | 0.17 | 0.02 | 11.38 | < 0.001 |
|  | Hour S5 | -0.03 | 0.01 | -4.24 | < 0.001 |
|  | Julian Date S1 | 0.18 | 0.01 | 15.05 | < 0.001 |
|  | Julian Date S2 | 0.77 | 0.01 | 123.08 | < 0.001 |
|  | Sex: male | -0.05 | 0.11 | -0.45 | 0.66 |
|  | Wolf RSF S1 | -0.02 | 0.50 | -0.05 | 0.96 |
|  | Wolf RSF S2 | -3.45 | 0.89 | -3.86 | < 0.001 |
| VNP | Intercept | 0.77 | 0.28 | 2.71 | 0.009 |
|  | Hour S1 | -0.45 | 0.01 | -44.50 | < 0.001 |
|  | Hour S2 | -0.55 | 0.01 | -45.15 | 0.2712 |
|  | Hour S3 | -0.13 | 0.01 | -12.91 | < 0.001 |
|  | Hour S4 | -0.49 | 0.02 | -27.76 | < 0.001 |
|  | Hour S5 | 0.11 | 0.01 | 13.84 | < 0.001 |
|  | Julian Date S1 | 0.15 | 0.02 | 9.13 | < 0.001 |
|  | Julian Date S2 | 0.28 | 0.01 | 36.63 | < 0.001 |
|  | Sex: male | 0.10 | 0.13 | 0.75 | 0.46 |
|  | Wolf RSF S1 | -0.11 | 0.39 | -0.29 | 0.77 |
|  | Wolf RSF S2 | -4.11 | 1.27 | -3.23 | 0.003 |
|  | Fix interval: 20 min | 0.53 | 0.14 | 3.77 | < 0.001 |

*”S” stands for spline

**Figure S1.)** Predicted GPS-collared wolf pack resource selection in northeastern Minnesota (NEMN) and Voyageurs National Park ecosystem (VNP) during spring (April – June), summer (July – October), and winter (November – March. Maps were projected in UTM 15N.

**
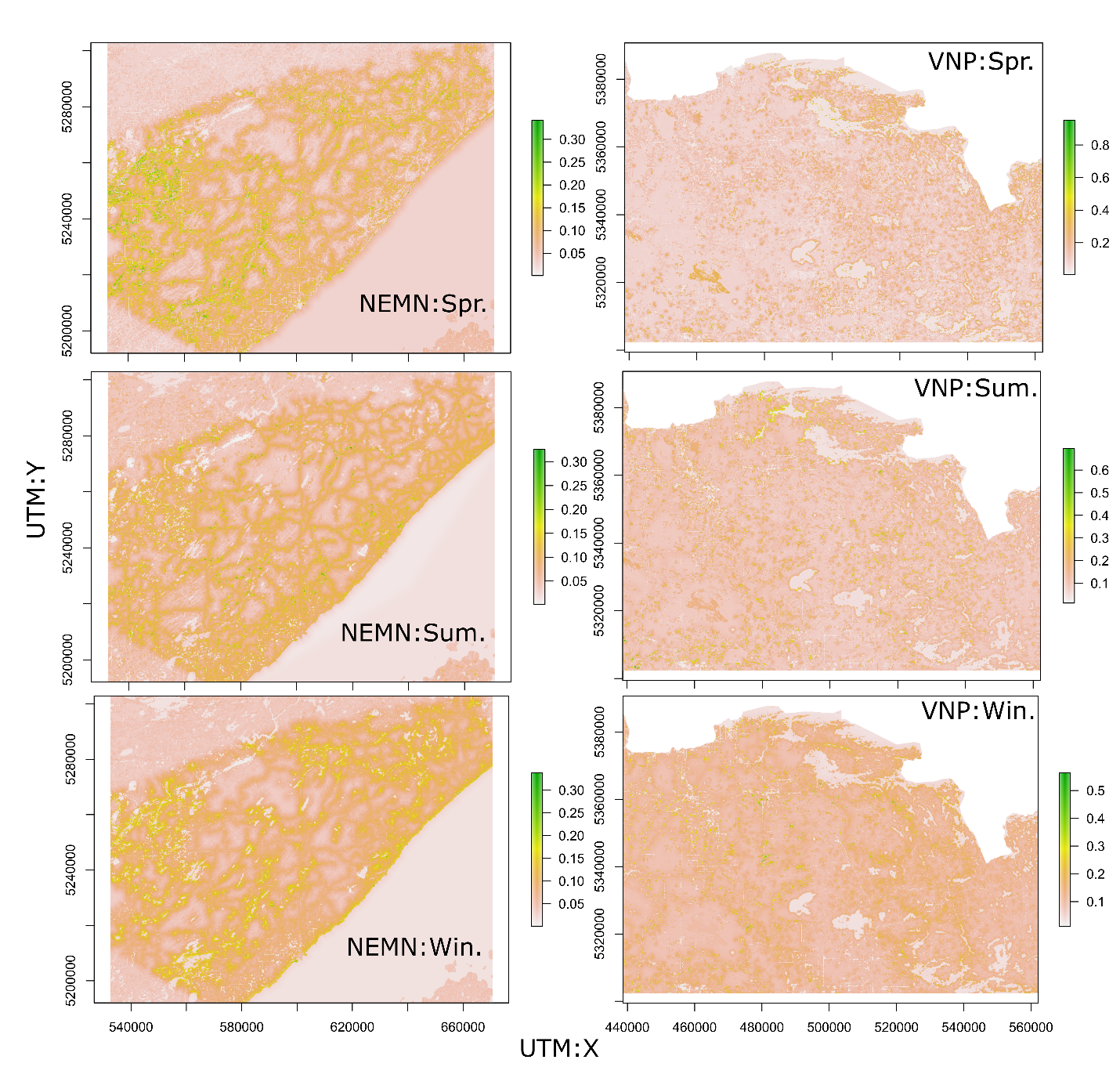
**

**Figure S2.)** Mean (95% confidence interval) first-passage time (in hours) of GPS-collared moose located in northeastern Minnesota and the Voyageurs National Park ecosystem by season and hour of the day. Seasons were delineated as spring = April – June; summer = July – October; winter = November – March. We calculated the mean FPT for each hour by averaging the mean FPT values of each moose-year.


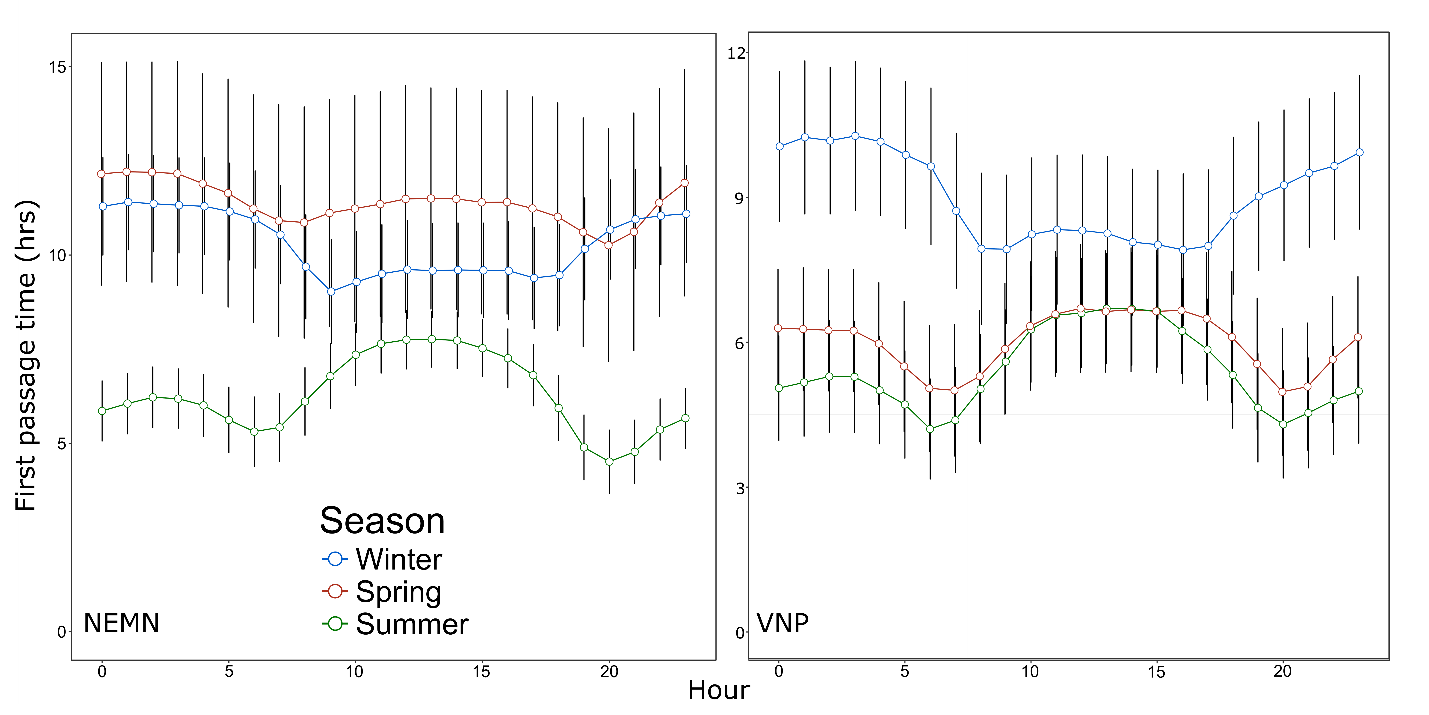


**Figure S3)** Results of a sensitivity analysis comparing the relationship between wolf RSF (scaled and centered) and moose FPT using ± 1 SE of the predicted wolf RSF values in our current model instead of the mean wolf RSF values found within each FPT radius. Black lines represent the mean response using the mean wolf RSF values (and 95% pointwise confidence intervals in gray), as was displayed in Figure 6, along with the mean estimates using ± 1 SE of the predicted wolf RSF values shown as red (-1 SE) and blue (+1 SE) lines. GPS-collared moose and wolves were located in the study areas of northeastern Minnesota (NEMN) and the Voyageurs National Park ecosystem (VNP). Seasons were defined as: spring = April – June, summer = July – October, and winter = November – March. We used linear mixed models to assess the influence of predicted wolf RSF values on moose FPT and we made predictions across the 99% quantile range of observed wolf RSF values in a given study area and season. Other continuous predictors were set to their mean values and categorical predictors were set to their mode except for fix rate which was set at 20 mins for easier comparisons between the study sites.


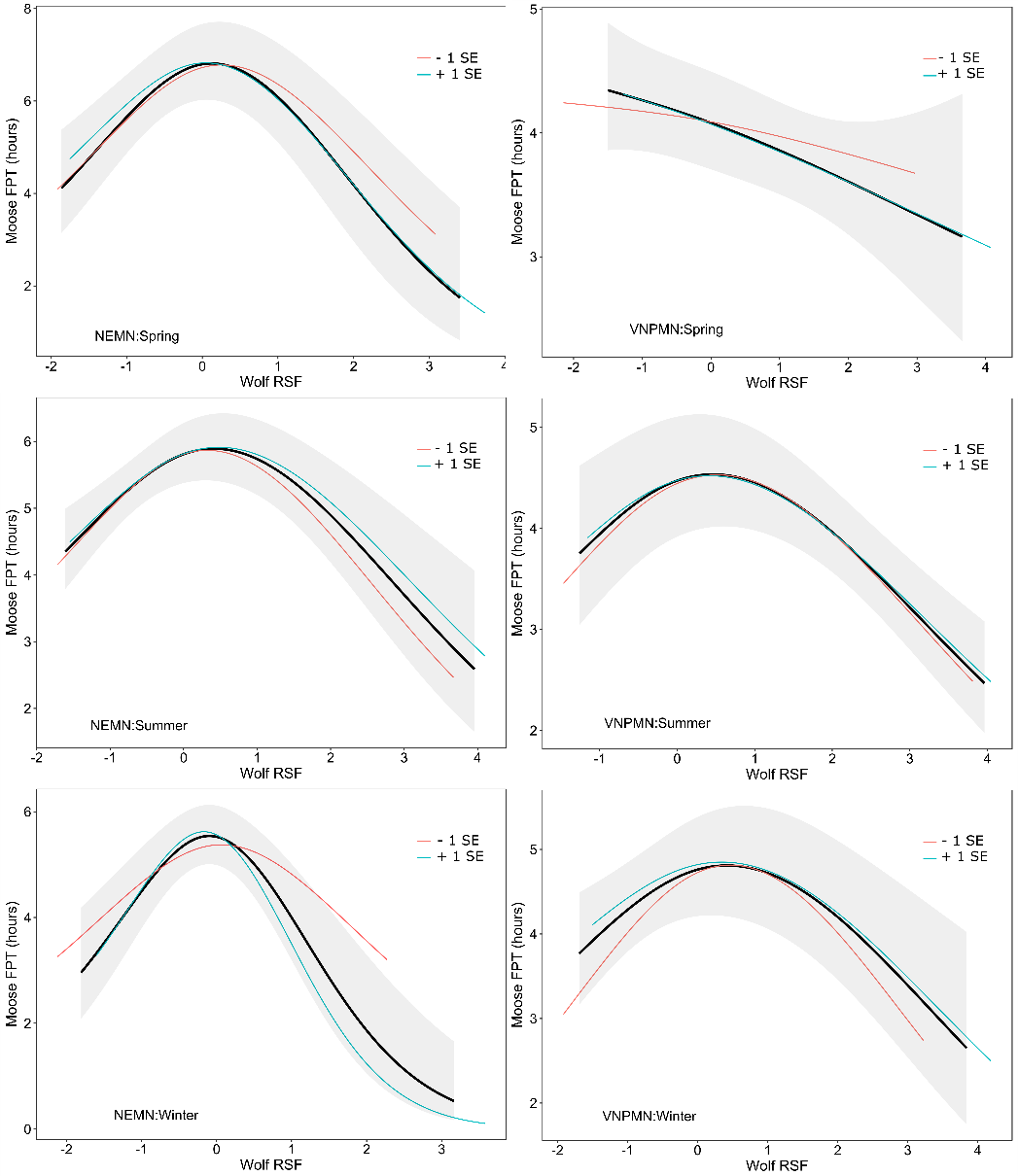

Supplement: Supplementary file 1 [file ECE3-8-9017-s001.docx]
